# Beyond the Horizon: Rethinking Prostate Cancer Treatment Through Innovation and Alternative Strategies

**DOI:** 10.3390/cancers17010075

**Published:** 2024-12-29

**Authors:** Siddhant Bhoir, Arrigo De Benedetti

**Affiliations:** 1 Department of Biochemistry and Molecular Biology, LSU Health Shreveport, Shreveport, LA 71103, USA; siddhantpandurang.bhoir@yale.edu; 2 Department of Therapeutic Radiology, School of Medicine, Yale University, 15 York Street, New Haven, CT 06510, USA

**Keywords:** prostate cancer (PCa), androgen receptor signaling, tousled-like kinase 1 (TLK1) pathway inhibition, TLK1 inhibitor, J54

## Abstract

For nearly a century, fundamental observations that prostate cancer (PCa) cells nearly always require AR stimulation for sustained proliferation have led to a unidirectional quest to abrogate such a pathway. Similarly focused have been efforts to understand AR-driven processes in the context of elevated expression of its target genes, and much less so on products that become overexpressed when AR signaling is suppressed. Treatment with ARSI results in an increased expression of the TLK1B splice variant via a ‘translational’ derepression driven by the compensatory mTOR activation and consequent activation of the TLK1 > NEK1 > ATR > Chk1 and NEK1 > YAP axes. In due course, this results first in a pro-survival quiescence and then adaptation to ADT and CRPC progression. This constitutes a novel liability for PCa that we have targeted for several years and novel approaches.

## Simple Summary

Prostate cancer (PCa) cells rely on androgen receptor (AR) signaling, and therapies targeting this pathway can inadvertently activate compensatory mechanisms like mTOR. This drives TLK1B overexpression, promoting survival and progression to castration-resistant prostate cancer (CRPC) via NEK1 > ATR > Chk1 and NEK1 > YAP pathways. Targeting this adaptation presents a novel therapeutic opportunity for CRPC.

## 1. Introduction

**Figure 1:**
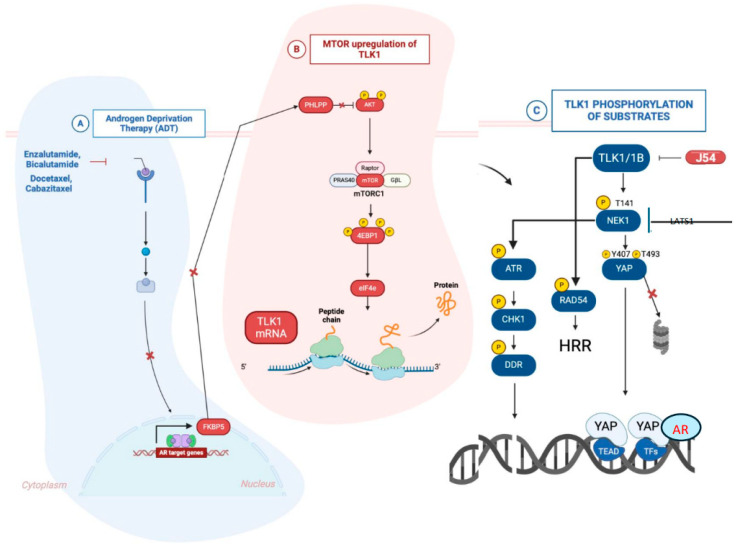
Model of TLK1B mTOR-driven translational derepression, followed by phosphorylation and activation of some of its key targets.

PCa relapse, a significant cause of cancer-related deaths in men, underscores the pressing need for alternative methodologies. Despite improvements in diagnostics and treatments, the leading standards of care—primarily androgen deprivation therapy (ADT), shown in Figure 1, and androgen-receptor signaling inhibitors (ARSI)—have not seen significant improvement for many years. This stagnation fails to address the immediate need for alternative methods to combat the diverse biological landscapes of this malignancy. While ADT and ARSI can temporarily control cancer progression by targeting androgen-driven pathways, they do not provide a definitive cure. Moreover, they are associated with severe adverse effects such as cardiovascular issues, metabolic disturbances, and cognitive decline. The emergence of resistance to ADT and ARSI further underscores the critical need for innovative therapies to overcome current limitations. This commentary delves into the importance of exploring alternative strategies in PCa treatment. It highlights some of our groundbreaking research at LSU Health Shreveport on Tousled-like kinase 1 (TLK1) and the J54 drug test molecule that could be poised to revolutionize our therapeutic approach, offering hope for a brighter future in PCa treatment [1].

## 2. History of Androgen Receptor Targeting

The history of androgen receptor (AR) targeting in prostate cancer (PCa) began with the landmark discovery by Charles Huggins and Clarence V. Hodges in 1941, showing that androgen deprivation, through orchiectomy or synthetic estrogens like stilbestrol, reduced levels of acid phosphatase, an early PCa biomarker [2]. This established androgen dependency in prostate tumors and laid the foundation for AR-targeted therapies.

The development of steroidal anti-androgens, such as cyproterone acetate (CPA), introduced AR inhibition by competitively blocking receptor activity while suppressing androgen synthesis and spermatogenesis. However, CPA’s mixed agonist–antagonist effects and off-target interactions with other steroid receptors limited its use due to side effects [3,4,5,6,7,8,9,10,11,12]. The introduction of non-steroidal anti-androgens like flutamide was a significant advancement. These agents effectively blocked testosterone and dihydrotestosterone (DHT) binding to AR, inhibiting receptor-mediated gene regulation and tumor growth. Despite their promise, these treatments disrupted feedback regulation of testosterone at the hypothalamus and pituitary, resulting in systemic effects [13,14,15].

Resistance has been a persistent challenge in AR-targeted therapy. AR mutations that alter drug binding can reduce efficacy, while the phenomenon of anti-androgen withdrawal—where stopping treatment temporarily regresses disease—highlights the complexity of AR signaling in PCa [13,14,15]. Recent discoveries have revealed AR’s structural and functional versatility. It can exist as monomers, dimers, or higher-order oligomers, even in the absence of ligands [12,16,17]. These findings underscore AR’s dynamic interactions with co-regulatory proteins and its central role in PCa progression. Addressing these complexities requires refined therapeutic approaches to overcome resistance and improve treatment outcomes. This historical perspective demonstrates the ongoing evolution of AR-targeted strategies, reflecting both breakthroughs in understanding AR biology and the challenges in addressing PCa’s adaptive mechanisms.

## 3. Reimagining the Treatment Paradigm: Moving Beyond Hormonal Manipulation

ADT has been the mainstay of PCa treatment for many years [18]. Its ability to reduce tumor size and slow disease progression is well documented but not a cure-all. As tumors progress into metastatic castration-resistant PCa (mCRPC), a more aggressive and treatment-resistant form, the effectiveness of ADT diminishes. The dominance of ADT [and use of potent ARSI’s such as abiraterone, bicalutamide, enzalutamide, apalutamide, and darolutamide] has led to a narrow focus in research and development, focusing on fine-tuning hormonal manipulation rather than exploring alternative new possibilities. This unidirectional approach limits expected patient outcomes and stifles our scientific creativity. The real challenge is to break free from this therapeutic monotony and invest in innovative approaches that target alternative pathways in PCa progression. The future of PCa care demands an urgent shift in focus from merely controlling the disease to dismantling its complex molecular architecture. 

## 4. Linking Tousled-like Kinase 1 to Prostate Cancer

A significant advancement in PCa research has emerged from our lab’s decade-long investigation into the role of Tousled-like kinase 1 (TLK1). TLK1 is a crucial regulator of PCa cells’ adaptation to ADT, driving androgen-independent growth, inhibiting apoptosis, and promoting metastasis. Our findings, summarized and published in the *International Journal of Molecular Sciences*, demonstrate that inhibiting TLK1 could significantly delay or prevent progression to mCRPC, offering a promising new strategy to enhance current treatments and reduce resistance [1]. This marks a bold step in diversifying therapeutic options for PCa.

Building on these findings, we developed J54, a novel TLK1 inhibitor. Published in iScience and recently granted a U.S. patent [20240189321], J54 has shown dual-action potential in preclinical studies, impairing cancer cell survival and sensitizing tumors to existing treatments [19]. This approach holds promise for patients who have exhausted conventional therapies such as ADT and chemotherapy, offering new hope by enhancing treatment efficacy and reducing the likelihood of resistance.

Our earlier research in 2013 identified a few phenothiazine (PTH) antipsychotics that inhibit TLK1 [20]. Through screening 6000 compounds, thioridazine (THD) emerged as a potent inhibitor, demonstrating growth-inhibitory effects on different PCa cell lines when combined with genotoxins or ARSI. However, its anti-dopaminergic activity, linked to severe side effects like cardiac arrhythmia and extrapyramidal toxicity, necessitated the development of safer alternatives. In collaboration with medicinal chemists and structural modelers, we synthesized J54, a selective TLK1 inhibitor with minimal toxicity. Preliminary studies indicate that J54 weakly binds to dopamine receptors, significantly reducing the risk of side effects commonly associated with PTH compounds [19].

The standard therapeutic strategy of ADT combined with ARSI is to force quiescence onto PCa cells but not their demise. This is because the mechanism of G1 arrest via DNA damage and response (DDR) induction by essentially all anti-androgens is attributable to the role played by the androgen receptor (AR) as a replication licensing factor [21]. In contrast, our lab’s novel approach seeks to “disrupt” this critical survival-related cell cycle arrest by targeting the TLK1 > NEK1 > ATR > CHK1 pathway, thus driving apoptosis. By bypassing this arrest with J54, particularly under the replication stress caused by ARSI, PCa cells are driven into catastrophic mitosis. This mechanism is further compounded by the difficulty of compensating for the lack of AR activity through Nek1-driven YAP activation [22,23,24], which potentiates AR signaling when androgens are scarce or outcompeted. In sum, J54 offers a novel and effective therapeutic strategy, overcoming the limitations of existing treatments by promoting apoptosis and preventing resistance in advanced PCa.

## 5. Expanding Our Research for Broader Public Health Benefits

The implications of TLK1 research and the J54 molecule extend far beyond the laboratory. According to the latest genome-wide association studies (GWAS), PCa disproportionately affects African-American (AA) men, who are 1.7 times more likely to be diagnosed and 2.3 times more likely to die from the disease compared to their Caucasian (CC) counterparts. As discovered, CC PCa patients tend to have fewer gene mutations related to the nucleotide excision repair (NER) pathway [25]. This stark contrast necessitates reevaluating the standard treatment paradigm, which has thus far failed to adequately address the complex interplay of genetic, socioeconomic, and access-related factors contributing to these disparities. The emergence of alternative therapeutic strategies, such as J54, holds promise in mitigating these inequities by offering more effective and personalized treatment options that could significantly benefit this demographic. For instance, personalized treatments could exploit genetic liabilities and differences in the AA “repairome” to target chemotherapies more effectively.

Cells employ intricate DNA repair and tolerance mechanisms to manage damage from internal and external sources, with dysfunction in these pathways closely tied to cancer progression. NER repairs bulky, helix-distorting lesions. In contrast, more minor, non-distorting lesions and single-strand breaks (SSBs) are rectified through base excision repair (BER). Double-strand breaks (DSBs) are typically resolved via homologous recombination (HRR), an error-free process, or nonhomologous end-joining (NHEJ), which can introduce mutations. In recent work, we have identified the critical and multifunctional protein, RAD54, as also being regulated by TLK1 [26]. PCa cells often show elevated expression of HRR proteins like RAD51 and RAD54, which we discovered are spatiotemporally regulated directly by TLK1 during various HRR/DSB recovery steps [26]. In a recently published report, we also explored the possibility of using DNA lesion-inducing agents like cisplatin (CPT), which creates purine adducts and crosslinks, combined with inhibition of the TLK1 > RAD54 > HRR pathway via J54, and achieved synthetic lethality [27]. This approach leveraged the deficiencies in NER and the overreliance on HRR in PCa, particularly in AA populations, to target cancer cells more effectively. TLK1, a serine/threonine kinase upregulated in PCa under anti-androgen therapy, phosphorylates HRR proteins like RAD54, promoting repair of DSBs from IR and radiomimetics [26]. However, inhibiting TLK1 can transform these DSBs into a therapeutic vulnerability, leading to enhanced cancer cell death when paired with agents like CPT. This dual-drug strategy offers a promising path for overcoming CPT resistance [27] and advancing alternative treatments for PCa patients.

Our research on connecting TLK1 with PCa continues to evolve, and our efforts exemplify the powerful synergy between academic innovation and public support. The development and implementation of alternative treatments stand to yield substantial cost savings within the healthcare system, thus alleviating the economic burden of PCa on both patients and society at large. Therefore, a proactive support model is paramount in breaking the cycle of treatment stagnation and championing the next generation of cancer therapies. This approach offers a more sanguine outlook for the future, positioning us to navigate the challenges posed by PCa with renewed optimism.

## 6. Emerging Research on TLKs from Other Labs

TLKs (Tousled-like kinases), particularly TLK1 and TLK2, are crucial for DNA replication, repair, chromatin assembly, and transcription [28,29,30,31,32]. While TLK1 and TLK2 share overlapping roles in genome integrity, mutations in TLK2 have been linked to neurological disorders and intellectual disabilities [33,34]. Additionally, TLK2 is frequently amplified in estrogen receptor-positive (ER+) breast cancers, where its inhibition—alone or combined with tamoxifen—significantly reduced tumor growth in preclinical models, highlighting its potential as a therapeutic target [35].

Initially, Asf1 was thought to be the primary TLK substrate due to its role in chromatin assembly [34]. However, later studies revealed distinct differences between TLK and Asf1 knockdowns, with research showing that Asf1 phosphorylation was performed by DNA-PK, not TLK, following DNA damage. Moreover, TLK knockout animals remain viable and fertile, suggesting TLKs could be therapeutically targeted without severe developmental side effects [36].

TLK2 inhibitors were identified in earlier screenings, and while their potency and off-target effects remain unclear, they present an intriguing area of research. Studies have shown that non-specific inhibitors like staurosporine and indirubin derivatives can suppress TLK2. Some PTH compounds (perphenazine, trifluoperazine, and thioridazine) have shown significant growth inhibition in high TLK2-expressing breast cancer cell lines, while low TLK2-expressing lines were less affected [35,37,38].

A derivative compound, J54, obtained in our custom syntheses, inhibits the TLK1-NEK1-ATR-Chk1 DDR pathway and induces apoptosis when combined with ARSI with high specificity in PCa cells. In comparison with a similar strategy, ATRi+Darolutamide was at best cytostatic with LAPC4 xenografts [39], likely because of the limited range of ATRi before generalized toxicity.

In summary, PTH derivatives demonstrate promise as cancer therapies targeting TLK1/2-mediated DDR pathways. Moreover, the findings from our lab and others highlight the crucial role TLKs play in cancer biology, generating growing interest and further exploration in this field.

## 7. Other New Approaches to AR Inhibition in CRPC and Associated Clinical Trials

Clinical trials such as STAMPEDE [Systemic Therapy in Advancing or Metastatic Prostate Cancer: Evaluation of Drug Efficacy (NCT00268476)] and LATITUDE are exploring the effectiveness of combining new therapeutic agents with existing treatments to improve outcomes in advanced PCa [40,41]. Early results suggest that adding anti-androgens to standard care can significantly improve survival, although managing the increased side effects remains a critical concern. Investigating new strategies that target different domains of AR is essential to overcoming resistance and improving the effectiveness of treatments.

### 7.1. Selective Androgen Receptor Degraders (SARDs)

Selective androgen receptor degraders (SARDs) present a promising new approach by promoting the degradation of AR [42,43], potentially overcoming resistance caused by mutations. Preclinical studies have shown SARDs are effective against resistant PCa models, suggesting their potential as a therapeutic strategy. Continued research into the mechanisms of action of SARDs is vital to developing treatment options for patients with advanced PCa.

### 7.2. The AR Amino-Terminal Domain

The AR amino-terminal domain (NTD) has emerged as a target for novel inhibitors, with recent studies identifying compounds that exhibit anti-androgen activity and reduce tumor burden [44]. High-throughput screening and marine-derived compounds are also being explored for their ability to inhibit AR function and improve treatment outcomes. Further investigation into NTD’s role in AR signaling is critical for developing effective therapies for PCa.

### 7.3. BF3 Surface Pocket

The AR’s BF3 surface pocket has been identified as a potential drug target, and small molecules show promise in modulating receptor activity [45,46]. While no compounds targeting this pocket have yet reached clinical trials, research is ongoing to explore its therapeutic potential in PCa treatment.

### 7.4. The DNA Binding Domain

The AR’s DNA-binding domain (DBD) has been a less-targeted area for drug development due to its similarity to other steroid receptors. However, new approaches, such as PROTACs, show promise. Small-molecule inhibitors targeting the DBD have demonstrated tissue-specific activity, opening the door for selective therapies [47,48,49]. Further exploration of DBD-targeting strategies is necessary to expand treatment options for PCa.

### 7.5. The Future of Anti-Androgen Therapy

The future of anti-androgen therapy lies in targeting multiple AR domains and developing novel inhibitors (such as J54) to counteract resistance mechanisms in advanced PCa. The AR NTD and other non-ligand-binding regions offer new avenues for drug discovery to improve patient outcomes. Although progress is being made, translating these novel therapies into clinical practice remains challenging, necessitating continued research and innovation.

## 8. Redefining the Future of Prostate Cancer Care

The research on TLK1/2 and the development of J54 foregrounds the critical need to rethink our approach to PCa treatment. It is vital to move beyond incremental modifications of current therapeutic approaches and forge novel strategies that address the disease from fresh angles. Such an undertaking necessitates a collective paradigm shift among clinicians, researchers, policymakers, and patients toward embracing the intricate nature of cancer biology and its manifold therapeutic prospects. Only by doing so can we significantly progress in the fight against PCa. This collective shift in mindset is not just a philosophical change but a practical necessity to foster collaboration, innovation, and progress in PCa treatment. Our work offers a glimpse into what is possible when prioritizing innovation over convention. As we stand on the cusp of a new era in PCa care, it is imperative to champion research that dares to defy the status quo. The future of treatment lies not in perfecting old tools but in forging new ones that offer hope, efficacy, and equity for all.

## 9. Conclusions

PCa treatment is at a critical juncture, with traditional therapies like ADT and ARSIs offering only temporary solutions. The emergence of resistance and severe side effects necessitate the exploration of innovative strategies beyond hormonal manipulation. Our research on Tousled-like kinase 1 (TLK1) and the development of J54 represent promising breakthroughs, targeting the TLK1-NEK1-ATR-Chk1 and NEK1 > YAP pathways to drive apoptosis and prevent resistance in advanced PCa. Coupled with advancements in AR inhibition, such as SARDs and new inhibitors targeting different AR domains, these novel approaches offer the potential to redefine PCa treatment. By integrating these strategies, we can overcome current limitations, enhance therapeutic efficacy, and provide hope for improved patient outcomes, particularly in high-risk groups. Continued research and clinical translation are essential for realizing these innovations and shaping the future of PCa care.

## Data Availability

Not Applicable.

## References

[1] Bhoir S., De Benedetti A. (2023). Targeting Prostate Cancer, the ‘Tousled Way’. Int. J. Mol. Sci..

[2] Huggins C., Hodges C.V. (1972). Studies on prostatic cancer. I. The effect of castration, of estrogen and androgen injection on serum phosphatases in metastatic carcinoma of the prostate. CA Cancer J. Clin..

[3] van Royen M.E., Cunha S.M., Brink M.C., Mattern K.A., Nigg A.L., Dubbink H.J., Verschure P.J., Trapman J., Houtsmuller A.B. (2007). Compartmentalization of androgen receptor protein-protein interactions in living cells. J. Cell Biol..

[4] Wang Q., Li W., Liu X.S., Carroll J.S., Janne O.A., Keeton E.K., Chinnaiyan A.M., Pienta K.J., Brown M. (2007). A hierarchical network of transcription factors governs androgen receptor-dependent prostate cancer growth. Mol. Cell.

[5] Yu X., Yi P., Hamilton R.A., Shen H., Chen M., Foulds C.E., Mancini M.A., Ludtke S.J., Wang Z., O’Malley B.W. (2020). Structural Insights of Transcriptionally Active, Full-Length Androgen Receptor Coactivator Complexes. Mol. Cell.

[6] Powell S.M., Christiaens V., Voulgaraki D., Waxman J., Claessens F., Bevan C.L. (2004). Mechanisms of androgen receptor signalling via steroid receptor coactivator-1 in prostate. Endocr. Relat. Cancer.

[7] Cano L.Q., Lavery D.N., Bevan C.L. (2013). Mini-review: Foldosome regulation of androgen receptor action in prostate cancer. Mol. Cell. Endocrinol..

[8] Reebye V., Querol Cano L., Lavery D.N., Brooke G.N., Powell S.M., Chotai D., Walker M.M., Whitaker H.C., Wait R., Hurst H.C. (2012). Role of the HSP90-associated cochaperone p23 in enhancing activity of the androgen receptor and significance for prostate cancer. Mol. Endocrinol..

[9] Shatkina L., Mink S., Rogatsch H., Klocker H., Langer G., Nestl A., Cato A.C. (2003). The cochaperone Bag-1L enhances androgen receptor action via interaction with the NH_2_-terminal region of the receptor. Mol. Cell. Biol..

[10] Yong W., Yang Z., Periyasamy S., Chen H., Yucel S., Li W., Lin L.Y., Wolf I.M., Cohn M.J., Baskin L.S. (2007). Essential role for Co-chaperone Fkbp52 but not Fkbp51 in androgen receptor-mediated signaling and physiology. J. Biol. Chem..

[11] Nadal M., Prekovic S., Gallastegui N., Helsen C., Abella M., Zielinska K., Gay M., Vilaseca M., Taules M., Houtsmuller A.B. (2017). Structure of the homodimeric androgen receptor ligand-binding domain. Nat. Commun..

[12] Presman D.M., Hager G.L. (2017). More than meets the dimer: What is the quaternary structure of the glucocorticoid receptor?. Transcription.

[13] Habenicht U.F., Schroder H., el Etreby M.F., Neumann F. (1988). Advantages and disadvantages of pure antiandrogens and of antiandrogens of the cyproterone acetate-type in the treatment of prostatic cancer. Prog. Clin. Biol. Res..

[14] Torri V., Floriani I. (2005). Cyproterone acetate in the therapy of prostate carcinoma. Arch. Ital. Urol. Androl..

[15] Raynaud J.P., Bonne C., Moguilewsky M., Lefebvre F.A., Belanger A., Labrie F. (1984). The pure antiandrogen RU 23908 (Anandron), a candidate of choice for the combined antihormonal treatment of prostatic cancer: A review. Prostate.

[16] Presman D.M., Ganguly S., Schiltz R.L., Johnson T.A., Karpova T.S., Hager G.L. (2016). DNA binding triggers tetramerization of the glucocorticoid receptor in live cells. Proc. Natl. Acad. Sci. USA.

[17] Jimenez-Panizo A., Perez P., Rojas A.M., Fuentes-Prior P., Estebanez-Perpina E. (2019). Non-canonical dimerization of the androgen receptor and other nuclear receptors: Implications for human disease. Endocr. Relat. Cancer.

[18] Litvinov I.V., De Marzo A.M., Isaacs J.T. (2003). Is the Achilles’ heel for prostate cancer therapy a gain of function in androgen receptor signaling?. J. Clin. Endocrinol. Metab..

[19] Singh V., Bhoir S., Chikhale R.V., Hussain J., Dwyer D., Bryce R.A., Kirubakaran S., De Benedetti A. (2020). Generation of Phenothiazine with Potent Anti-TLK1 Activity for Prostate Cancer Therapy. iScience.

[20] Ronald S., Awate S., Rath A., Carroll J., Galiano F., Dwyer D., Kleiner-Hancock H., Mathis J.M., Vigod S., De Benedetti A. (2013). Phenothiazine Inhibitors of TLKs Affect Double-Strand Break Repair and DNA Damage Response Recovery and Potentiate Tumor Killing with Radiomimetic Therapy. Genes Cancer.

[21] Litvinov I.V., Vander Griend D.J., Antony L., Dalrymple S., De Marzo A.M., Drake C.G., Isaacs J.T. (2006). Androgen receptor as a licensing factor for DNA replication in androgen-sensitive prostate cancer cells. Proc. Natl. Acad. Sci. USA.

[22] Ghosh I., Khalil M.I., Mirza R., King J., Olatunde D., De Benedetti A. (2023). NEK1-Mediated Phosphorylation of YAP1 Is Key to Prostate Cancer Progression. Biomedicines.

[23] Khalil M.I., Ghosh I., Singh V., Chen J., Zhu H., De Benedetti A. (2020). NEK1 Phosphorylation of YAP Promotes Its Stabilization and Transcriptional Output. Cancers.

[24] Olatunde D., De Benedetti A. (2024). TLK1>Nek1 Axis Promotes Nuclear Retention and Activation of YAP with Implications for Castration-Resistant Prostate Cancer. Cancers.

[25] Yadav S., Anbalagan M., Baddoo M., Chellamuthu V.K., Mukhopadhyay S., Woods C., Jiang W., Moroz K., Flemington E.K., Makridakis N. (2020). Somatic mutations in the DNA repairome in prostate cancers in African Americans and Caucasians. Oncogene.

[26] Ghosh I., Kwon Y., Shabestari A.B., Chikhale R., Chen J., Wiese C., Sung P., De Benedetti A. (2023). TLK1-mediated RAD54 phosphorylation spatio-temporally regulates Homologous Recombination Repair. Nucleic Acids Res..

[27] Bhoir S., Ogundepo O., Yu X., Shi R., De Benedetti A. (2023). Exploiting TLK1 and Cisplatin Synergy for Synthetic Lethality in Androgen-Insensitive Prostate Cancer. Biomedicines.

[28] Han Z., Riefler G.M., Saam J.R., Mango S.E., Schumacher J.M. (2005). The *C. elegans* Tousled-like kinase contributes to chromosome segregation as a substrate and regulator of the Aurora B kinase. Curr. Biol..

[29] Han Z., Saam J.R., Adams H.P., Mango S.E., Schumacher J.M. (2003). The *C. elegans* Tousled-like kinase (TLK-1) has an essential role in transcription. Curr. Biol..

[30] Carrera P., Moshkin Y.M., Gronke S., Sillje H.H., Nigg E.A., Jackle H., Karch F. (2003). Tousled-like kinase functions with the chromatin assembly pathway regulating nuclear divisions. Genes Dev..

[31] De Benedetti A. (2012). The Tousled-Like Kinases as Guardians of Genome Integrity. ISRN Mol. Biol..

[32] Klimovskaia I.M., Young C., Stromme C.B., Menard P., Jasencakova Z., Mejlvang J., Ask K., Ploug M., Nielsen M.L., Jensen O.N. (2014). Tousled-like kinases phosphorylate Asf1 to promote histone supply during DNA replication. Nat. Commun..

[33] Pavinato L., Villamor-Paya M., Sanchiz-Calvo M., Andreoli C., Gay M., Vilaseca M., Arauz-Garofalo G., Ciolfi A., Bruselles A., Pippucci T. (2022). Functional analysis of TLK2 variants and their proximal interactomes implicates impaired kinase activity and chromatin maintenance defects in their pathogenesis. J. Med. Genet..

[34] Segura-Bayona S., Stracker T.H. (2019). The Tousled-like kinases regulate genome and epigenome stability: Implications in development and disease. Cell. Mol. Life Sci..

[35] Kim J.A., Tan Y., Wang X., Cao X., Veeraraghavan J., Liang Y., Edwards D.P., Huang S., Pan X., Li K. (2016). Comprehensive functional analysis of the tousled-like kinase 2 frequently amplified in aggressive luminal breast cancers. Nat. Commun..

[36] Segura-Bayona S., Knobel P.A., Gonzalez-Buron H., Youssef S.A., Pena-Blanco A., Coyaud E., Lopez-Rovira T., Rein K., Palenzuela L., Colombelli J. (2017). Differential requirements for Tousled-like kinases 1 and 2 in mammalian development. Cell Death Differ..

[37] Gao Y., Davies S.P., Augustin M., Woodward A., Patel U.A., Kovelman R., Harvey K.J. (2013). A broad activity screen in support of a chemogenomic map for kinase signalling research and drug discovery. Biochem. J..

[38] Lin C.L., Tan X., Chen M., Kusi M., Hung C.N., Chou C.W., Hsu Y.T., Wang C.M., Kirma N., Chen C.L. (2020). ERalpha-related chromothripsis enhances concordant gene transcription on chromosome 17q11.1-q24.1 in luminal breast cancer. BMC Med. Genom..

[39] Wengner A.M., Siemeister G., Lücking U., Lefranc J., Wortmann L., Lienau P., Bader B., Bömer U., Moosmayer D., Eberspächer U. (2020). The Novel ATR Inhibitor BAY 1895344 Is Efficacious as Monotherapy and Combined with DNA Damage–Inducing or Repair–Compromising Therapies in Preclinical Cancer Models. Mol. Cancer Ther..

[40] James N.D., de Bono J.S., Spears M.R., Clarke N.W., Mason M.D., Dearnaley D.P., Ritchie A.W.S., Amos C.L., Gilson C., Jones R.J. (2017). Abiraterone for Prostate Cancer Not Previously Treated with Hormone Therapy. N. Engl. J. Med..

[41] Fizazi K., Tran N., Fein L., Matsubara N., Rodriguez-Antolin A., Alekseev B.Y., Ozguroglu M., Ye D., Feyerabend S., Protheroe A. (2019). Abiraterone acetate plus prednisone in patients with newly diagnosed high-risk metastatic castration-sensitive prostate cancer (LATITUDE): Final overall survival analysis of a randomised, double-blind, phase 3 trial. Lancet Oncol..

[42] Shibata N., Nagai K., Morita Y., Ujikawa O., Ohoka N., Hattori T., Koyama R., Sano O., Imaeda Y., Nara H. (2018). Development of Protein Degradation Inducers of Androgen Receptor by Conjugation of Androgen Receptor Ligands and Inhibitor of Apoptosis Protein Ligands. J. Med. Chem..

[43] Rodriguez-Gonzalez A., Cyrus K., Salcius M., Kim K., Crews C.M., Deshaies R.J., Sakamoto K.M. (2008). Targeting steroid hormone receptors for ubiquitination and degradation in breast and prostate cancer. Oncogene.

[44] Andersen R.J., Mawji N.R., Wang J., Wang G., Haile S., Myung J.K., Watt K., Tam T., Yang Y.C., Banuelos C.A. (2010). Regression of castrate-recurrent prostate cancer by a small-molecule inhibitor of the amino-terminus domain of the androgen receptor. Cancer Cell.

[45] Lallous N., Leblanc E., Munuganti R.S., Hassona M.D., Nakouzi N.A., Awrey S., Morin H., Roshan-Moniri M., Singh K., Lawn S. (2016). Targeting Binding Function-3 of the Androgen Receptor Blocks Its Co-Chaperone Interactions, Nuclear Translocation, and Activation. Mol. Cancer Ther..

[46] Estebanez-Perpina E., Arnold L.A., Nguyen P., Rodrigues E.D., Mar E., Bateman R., Pallai P., Shokat K.M., Baxter J.D., Guy R.K. (2007). A surface on the androgen receptor that allosterically regulates coactivator binding. Proc. Natl. Acad. Sci. USA.

[47] Li H., Ban F., Dalal K., Leblanc E., Frewin K., Ma D., Adomat H., Rennie P.S., Cherkasov A. (2014). Discovery of small-molecule inhibitors selectively targeting the DNA-binding domain of the human androgen receptor. J. Med. Chem..

[48] Lee G.T., Nagaya N., Desantis J., Madura K., Sabaawy H.E., Kim W.J., Vaz R.J., Cruciani G., Kim I.Y. (2021). Effects of MTX-23, a Novel PROTAC of Androgen Receptor Splice Variant-7 and Androgen Receptor, on CRPC Resistant to Second-Line Antiandrogen Therapy. Mol. Cancer Ther..

[49] Lim M., Otto-Duessel M., He M., Su L., Nguyen D., Chin E., Alliston T., Jones J.O. (2014). Ligand-independent and tissue-selective androgen receptor inhibition by pyrvinium. ACS Chem. Biol..

